# Supplementary material for: The genome sequence of the Little Owl, Athene noctua (Scopoli, 1769) (Strigiformes: Strigidae)
Source: Wellcome Open Res. 2025 Sep 8;10:492. [Version 1] doi: 10.12688/wellcomeopenres.24873.1 (PMC12616116; doi:10.12688/wellcomeopenres.24873.1)
Supplement: Peer review history

# Reviewer report: Reviewer response for version 1

**DOI:** 10.21956/wellcomeopenres.27397.r132129

**Published:** 2025-11-13

**Authors:** Rauri C.K. Bowie

**Affiliations:** 1 University of California Berkeley, Berkeley, USA

This data note presents a high-quality reference genome for the Little Owl. The methods are clearly detailed and meet the standards expected for a genome report. I have only a few minor comments for the authors to consider.

1. **Sample acquisition:** The authors should explicitly state whether a voucher (physical) specimen is available.  I assume it is, given that a Natural History Museum number – NHMUK015060570 – is reported, but this could be made clearer.  Are additional tissue samples available should someone want to perform future analyses on this individual?
2. **Assembly quality assessment**. What database did the BlobToolKit query for the BUSCO lineages?  The GoaT database? 
3. **Data availability**: Athene noctua should be in italics. 

Are sufficient details of methods and materials provided to allow replication by others?

Yes

Is the rationale for creating the dataset(s) clearly described?

Yes

Are the datasets clearly presented in a useable and accessible format?

Yes

Are the protocols appropriate and is the work technically sound?

Yes

Reviewer Expertise:

Evolutionary Biology, Genomics, Birds

I confirm that I have read this submission and believe that I have an appropriate level of expertise to confirm that it is of an acceptable scientific standard.

# Reviewer report: Reviewer response for version 1

**DOI:** 10.21956/wellcomeopenres.27397.r132126

**Published:** 2025-09-30

**Authors:** Marija Tanaskovic

**Affiliations:** 1 University of Belgrade, Beograd, Serbia

The manuscript describes the sequencing, assembly, and curation of a high-quality reference genome for *Athene noctua*. The authors apply state-of-the-art approaches, including PacBio HiFi sequencing, Hi-C scaffolding, and RNA-seq for annotation support. The assembly reaches Earth BioGenome Project (EBP) reference standards (6.C.Q62) with excellent continuity and completeness (scaffold N50 of 86.3 Mb, 97.3% BUSCO completeness, QV > 62). The work is clearly presented and will be of value to the community as an important resource for avian genomics and conservation biology.

 The study is technically sound, methodologically detailed, and reproducible. However, there are several areas where additional clarification and standardization of reporting would improve the manuscript’s clarity and alignment with current best practices.

**Major Comments**

1. **Sequencing depth reporting** PacBio coverage is reported (55×), but Hi-C and RNA-seq depth are not explicitly linked to genome size or downstream use. For Hi-C, please provide approximate coverage relative to genome size. For RNA-seq, indicate total yield (e.g., millions of reads per library) to support downstream annotation.
2. **Haplotype handling** The assembly strategy generates two haplotypes, with haplotype 1 curated to chromosome level and haplotype 2 scaffold-level. Please state explicitly which haplotype is considered the reference assembly for submission and downstream use, and how the second haplotype will be provided.
3. **Contamination assessment** Methods describe the use of ASCC and BlobToolKit for contamination screening, but results are not reported. Please summarize contamination screening outcomes in the results (e.g., confirmation of minimal non-target sequences).
4. **Assembly curation** Manual curation involved 19 breaks and 162 joins. Please provide a brief explanation of the types of misassemblies detected (e.g., chimeric joins, collapsed repeats) and the criteria for corrections, as this adds confidence in the final assembly.
5. **Minor Comments**
  - **QC metrics** QC reporting is inconsistent across DNA, RNA, and library preparations. For RNA, integrity values (RIN/RQN) should be reported, as they are provided for DNA purity and fragment size. For libraries, fragment size distributions and molarity should be reported uniformly.
  - **Redundancy in phrasing** The phrase “following the manufacturer’s instructions” is repeated frequently. This could be streamlined to improve readability (e.g., state once per subsection).
  - **Reference consistency **References to Rhie et al. (2021) and EBP standards should be formatted consistently. Ensure all software tools and pipelines (e.g., Qubit software version, TapeStation kits) are cited with versions 
**Recommendation M** **inor revisions** The genome assembly is of very high quality and meets the standards for publication as a reference genome. The manuscript requires only modest revisions for clarity, consistency, and completeness in reporting QC metrics, sequencing depth, contamination screening results, and haplotype designation

Are sufficient details of methods and materials provided to allow replication by others?

Yes

Is the rationale for creating the dataset(s) clearly described?

Yes

Are the datasets clearly presented in a useable and accessible format?

Yes

Are the protocols appropriate and is the work technically sound?

Yes

Reviewer Expertise:

population genetics

I confirm that I have read this submission and believe that I have an appropriate level of expertise to confirm that it is of an acceptable scientific standard.
